# Development of solid agents of the diphenyl ether herbicide degrading bacterium *Bacillus* sp. Za based on a mixed organic fertilizer carrier

**DOI:** 10.3389/fmicb.2022.1075930

**Published:** 2022-11-24

**Authors:** Guoqiang Zhao, Yanning Tian, Houyu Yu, Jintao Li, Dongmei Mao, Rayan Mazin Faisal, Xing Huang

**Affiliations:** ^1^Department of Microbiology, College of Life Sciences, Nanjing Agricultural University, Nanjing, China; ^2^Department of Biology, College of Science, University of Mosul, Mosul, Iraq

**Keywords:** *Bacillus* sp. Za, diphenyl ether herbicides, solid agents, organic fertilizer, soil remediation

## Abstract

The long-term and widespread use of diphenyl ether herbicides has caused serious soil residue problems and threatens the agricultural ecological environment. The development of biodegrading agents using high-efficiency degrading strains as pesticide residue remediation materials has been widely recognized. In this study, the strain *Bacillus* sp. Za was used to prepare solid agents for the remediation of diphenyl ether herbicides-contaminated soil. The ratio of organic fertilizer was 1:3 (pig manure: cow dung), the inoculum amount of Za was 10%, the application amount of solid agents was 7%, and the application mode was mixed application, all of which were the most suitable conditions for solid agents. After the solid agents were stored for 120 days, the amount of Za remained above 10^8^ CFU/g. The degradation rates of the solid agents for lactofen, bifenox, fluoroglycofen, and fomesafen in soil reached 87.40, 82.40, 78.20, and 65.20%, respectively, on the 7th day. The application of solid agents alleviated the toxic effect of lactofen residues on maize seedlings. A confocal laser scanning microscope (CLSM) was used to observe the colonization of Za-*gfp* on the surface of maize roots treated in the solid agents, and Za-*gfp* mainly colonized the elongation zone and the mature area of maize root tips, and the colonization time exceeded 21 days. High-throughput sequencing analysis of soil community structural changes in CK, J (solid agents), Y (lactofen), and JY (solid agents + lactofen) groups showed that the addition of solid agents could restore the bacterial community structure in the rhizosphere soil of maize seedlings. The development of solid agents can facilitate the remediation of soil contaminated with diphenyl ether herbicide residues and improve the technical level of the microbial degradation of pesticide residues.

## Introduction

As an important category of herbicide family, diphenyl ether herbicides have been widely studied and applied in the field of agriculture (Xie et al., 2018). They inhibit the porphyrin biosynthesis pathway of broadleaf weeds, giving rise to lipid peroxidative damage and membrane permeability damage in the cell membrane, resulting in the death of weeds (Scalla et al., 1990; Park et al., 2021). The main diphenyl ether herbicides are lactofen, fomesafen, oxyfluorfen, fluoroglycofen, aclonifen, acifluorfen, and bifenox. Generally, diphenyl ether herbicides are highly efficient, stable, pertinent and have been widely used for a long time in cereal crop and grain production areas in China (Diao et al., 2009). However, they are difficult to eliminate from the natural environment, and the issue of diphenyl ether herbicide residues in soil has become increasingly significant. The residue of these herbicides not only poison subsequent crops, such as maize, sorghum, wheat, cabbage, sugar beet, and flax, but also inhibit the activity of soil enzymes, ultimately affecting soil fertility and reducing crop yields (Mukherjee et al., 2007). In addition, they cause water pollution through surface leakage, with negative impacts on aquatic plants and animals (Wang et al., 2018).

Diphenyl ether herbicides have various degradation modes after entering the environment, including photolysis, hydrolysis, and microbial degradation (Singh and Singh, 2016). However, microbial degradation is mainly achieved through microbial activity, with a high removal effect. To date, many degrading strains that can catabolize diphenyl ether herbicides have been isolated. For example, 50 mg/ml fomesafen was shown to be effectively degraded (88.7%) by *Pseudomonas zeshuii* BY-1 within 3 days (Feng et al., 2012). *Pseudomonas* sp. FB8 and *Bacillus* sp. FE-1 were used to remediate fomesafen-contaminated soil (Yang et al., 2011; Cui et al., 2018), whereas *Brevundimonas* sp. LY-2 completed the biotransformation of lactofen (Liang et al., 2010). In another study, *Edaphocola flava* HME-24 degraded 96.7% of 50 mg/ml lactofen within 72 h (Hu et al., 2020). *Bacillus* sp. YS-1 was shown to degrade 50 mg/l lactofen at an efficiency rate that reached 97.6% within 15 h (Shang et al., 2022). Additionally, *Bacillus* sp. Za could degrade 94.8% of 50 mg/l lactofen within 4 days, and the phytotoxicity of the degradation product to maize was decreased significantly compared with that of lactofen (Zhang J. et al., 2018). These isolated bacteria provide strain resources for the remediation of sites contaminated with diphenyl ether herbicides.

Degradation bacteria are often easily affected by environmental factors, so it is difficult to play their environmental remediation function well (Krueger et al., 2015). However, the preparation of high-efficiency degrading strains into agents is considered to be an effective method (Galloway et al., 2017; Yuan et al., 2020). Bacterial agents can be divided into liquid and solid ones, depending on the dosage form (Zhu et al., 2019). However, liquid agents often have a short shelf life and show unstable effects (Wen et al., 2021). Compared with liquid agents, solid ones have many advantages, such as easy storage and transport, a longer action time, the protection of degrading bacteria from environmental influences, and the provision of a suitable microenvironment for bacterial survival (Ruan et al., 2018; Zhu et al., 2021). There have been some reports on the commercialization of high-efficiency degrading strains for the remediation of soil and water (Sayed and Behle, 2017a; Mohd Yusof et al., 2019). The solid agents prepared by the cyclic aromatic hydrocarbon-degrading bacteria greatly improved the degradation of the organic pollutant pyrene in soil (Wang et al., 2020). Using *Pseudomonas stutzeri* Y2 to prepare solid agents, the bacterial degradation of S-triazine herbicides in industrial wastewater and corn fields could be promoted (Zhang B. et al., 2020). The development of microbial agents has great application prospects in the degradation of environmental pollutants, and the application effect has been satisfactory. However, there are few reports on the development of bacterial agents for the degradation of diphenyl ether herbicides.

The microbial community structure of soil is often affected by various external factors. Some studies have shown that not only the long-term use of pesticides will affect the microbial community (Jacobsen and Hjelmsø, 2014), but that also the application of microbial agents will change the biodiversity and community structure (Li et al., 2022). Even though, different types of pesticides and microbial agents have different effects on the soil bacterial community structure, and the specific effects of pesticides and microbial agents on this structure need to be further analyzed.

In this study, the solid agents were prepared with the diphenyl ether-degrading bacterial strain *Bacillus* sp. Za isolated in our laboratory. The preparation process of the solid agents was optimized, and factors influencing phytotoxicity to maize by solid agents were explored. This study also analyzed the changes in the bacterial community structure after the application of solid agents in herbicide-contaminated soil. The results facilitate the development of herbicide-degrading strains, as well as future large-scale production and practical application.

## Materials and methods

### Chemicals, strains, and media

Lactofen, bifenox, fluoroglycofen, and fomesafen (analytical grade, purity > 99%) were purchased from Beijing Bailingwei Technology Co., Ltd. The HPLC-grade reagents were purchased from Sigma Aldrich, United States, and all other reagents were of analytical grade. Pig manure, as organic fertilizer, was purchased from Gansu Sudi Fertilizer Co., Ltd., and cow dung was obtained from Nanjing Sanmei Co., Ltd. The above-mentioned carrier was naturally air-dried for 3 d and passed through a 40-mesh sieve, followed by sterilization at 121°C for later use.

*Bacillus* sp. Za (Zhang J. et al., 2018) was preserved in our laboratory in LB medium with the following composition (g/L): 5.0 yeast extract, 10.0 peptone, and 10.0 NaCl. Mineral salts medium (MSM) contained (g/L): 1.0 NaCl, 1.5 K_2_HPO_4_, 0.5 KH_2_PO_4_, 1.0 NH_4_NO_3_, and 0.2 MgSO_4_.7H_2_O. Za fermentation medium contained (g/L): 25.74 molasses, 14.21 soy peptone, 3.0 NH_4_Cl, 3.5 K_2_HPO_4_, 1.0 KH_2_PO_4_, 2.0 NaCl, and 0.4 MgSO_4_⋅7H_2_O.

### Soil preparation and maize cultivation

Soil was collected from the Qiqiaoweng ecological wetland in Nanjing, which was loamy clay soil. At the site, no diphenyl ether herbicides had been applied, and the soil physical and chemical properties were as follows: pH 6.89, available P 14.36 mg/kg, available K 109.08 mg/kg, and organic matter 15.67 g/kg. The soil was naturally air-dried in a ventilated area, and the sundries were removed and passed through a 10-mesh sieve. According to the concentration of 10 mg/kg dry soil, lactofen, bifenox, fluoroglycofen, and fomesafen were added to the soil separately to obtain one portion of soil containing a single herbicide residue for use. The maize seeds (Meiyu No. 3, Jiangsu Tomorrow Seed Technology Co., Ltd.) were sterilized with 10% H_2_O_2_ solution and placed in the dark for germination, seeds with the same germination degree were selected for follow-up experiments (Qian et al., 2022).

### Preparation of solid agents of strain Za

Pig manure and cow dung were selected as carriers of solid bacterial agents, and a two-factor and three-level orthogonal experiment was designed according to the mixing ratio of the carrier and the inoculation amount of strain Za. The mixing ratio levels of pig manure and cow dung organic fertilizer were set as 3:1, 1:1, and 1:3, and the levels of strain Za inoculum were set as 5, 10, and 15%. The carriers were prepared according to the above carrier ratios, and 100 g of each was taken, sterilized at 121°C twice, and air-dried for later use. Under sterile conditions, the fermentation broth (the amount of Za reached 10^10^ CFU/ml) was inoculated into each carrier at inoculum levels of 5, 10, and 15%, and placed in a 30°C incubator for 3 days to check the viability, the average number of Za was taken as logarithmic treatment. Subsequently, weighed 5 g of solid agents treated in different treatments and added them to a sterilized conical flask containing 45 ml of sterile water, the plate method was used to detect the number of strain Za cells. The recovery rate of strain Za was the ratio between the Za after inoculation and the initial inoculation. According to the optimal mixture ratio and the optimal inoculum amount, the solid agents were prepared with different types of organic fertilizer carriers, then they were stored in a dry and cool place, and the dynamic changes in the Za count were observed within 120 days.

### Effects of the application amount and mode on the degradation of solid agents

The solid agents prepared and stored for 30 days (with the strain Za count of 10^9^ CFU/g) were applied to the soil containing 10 mg/kg lactofen, bifenox, fluoroglycofen, and fomesafen at 1, 3, 5, 7, and 9%, the water content was adjusted to 20% with deionized water and maintained. After 4 days, samples were taken to detect the residual amounts of the four herbicides in soil and calculate the degradation rate. The experiment was performed in three groups, and each treatment was placed in an incubator to simulate the natural environment.

Following the same procedure as described above, the solid agents were applied according to the optimal amount for covering application, mixed application, and acupoint application, respectively. Soil containing the herbicides without inoculation with the solid bacterial agent was used as control. On days 1, 3, 5, and 7, samples were taken to detect the residues of the four herbicides in soil.

### Relief effect of lactofen to maize phytotoxicity by solid agents and observation of Za-*gfp* colonization in maize rhizosphere

*Bacillus* sp. Za-*gfp* was constructed according to the method for labeling strains with GFP (Zhang et al., 2022), and used to prepare solid agents under optimal conditions. Soil containing the residues of lactofen was loaded into pots, and the optimal inoculum amount and application mode obtained were used to apply solid agents according to different treatments. The different treatments were set as follows: Control (without any addition), J (solid agents), Y5 (5 mg/kg lactofen), JY5 (5 mg/kg of lactofen + solid agents), Y10 (10 mg/kg of lactofen), and JY10 (10 mg/kg lactofen + solid agents). At the same time, the maize seeds with the same germination stage were picked and transplanted. The maize seedlings were sampled on the 7th, 14th, and 21st days, respectively, and physiological indicators such as stem and leaf length, root length, and fresh weight were measured. The maize seedling roots of groups Control and J (solid agents) were collected on the 7th, 14th, and 21st days for microscopic observation. At sampling, the root system was removed from the soil, gently shaken, and all soil particles were washed off from the root surface with sterile water, subsequently, the roots were cut into 0.5-cm-long parts with a clean blade. The root segments were divided into root cap, meristem, elongation area, and mature area, then they were fixed in a 48-well plate filled with 2.5% glutaraldehyde fixative, sliced on a glass slide, and observed under a confocal laser scanning microscope (CLSM, Leica TCS SP3). The excitation wavelength was 488 nm, and the collected signal range was 480–525 nm.

### Analysis of the soil community structure

The rhizosphere soil of maize seedlings was used as the research object. The collected seedlings were dug up with the root zone soil, and the root system was shaken gently to remove the soil that was weakly attached to the root system, the remaining soil was the rhizosphere soil. The maize seedlings of Control, J, Y10, and JY10 groups were selected as the research objects, and the sampling time points were set at days 0, 7, 14, and 21. The total soil DNA was extracted and sent to Shanghai Meiji Biomedical Technology Co., Ltd. for subsequent analysis by 16S rRNA amplification and sequencing of bacteria. The PE reads obtained by MiSeq sequencing were subjected to optimization steps such as quality control, filtering, and splicing. The Qiime2 process was used to analyze the microbial diversity, and the Dada2/Deblur noise reduction method was applied to obtain the representative sequence and abundance information of the Amplicon Sequence Variant. Based on the sequencing results, follow-up *Alpha* diversity analysis, *Beta* diversity analysis, community composition analysis, and species difference analysis were carried out (Wang et al., 2007).

### Chemical analysis

Lactofen, bifenox, fluoroglycofen, and fomesafen residues in soil were extracted with an equal volume of dichloromethane by shaking at 150 rpm for 1 h, and centrifugation at 8,000 × *g* for 10 min. All supernatants were collected and acidified to pH 2.5. The upper solution was discarded, the remaining solution was blown dry, dissolved in methanol, and analyzed by high-performance liquid chromatography (HPLC) (Dionex UltiMate 3,000). The chromatograph was equipped with a C18 reverse-phase chromatographic column (4.6 × 250 mm, 5 μm, Agilent Technologies, Palo Alto, CA, United States), the column temperature was 40°C, and the UV detector was set as 270 nm. The mobile phase was water: acetonitrile: phosphoric acid (40:60:0.5, v: v: v) and the flow rate was 1 ml/min.

### Data processing and analysis

One-way ANOVA and Duncan’s test to compare means were used to analyze the data. The statistically significant difference was set at *p*-values < 0.05. Using Microsoft Excel 2015 and IBM SPSS Statistics 20, the data were processed and analyzed for significant differences. Graphs were generated by the GraphPad Prism v. 8.0.2.263 software (San Diego, CA, USA).

### Data availability

All of the sequencing raw data involved in this manuscript had been deposited in the NCBI database (BioProject ID: PRJNA895898), and could be download from the link: https://www.ncbi.nlm.nih.gov/sra/PRJNA895898.

## Results

### Optimization of organic fertilizer carrier ratio and Za inoculum amount in the preparation of solid agents

**FIGURE 1:**
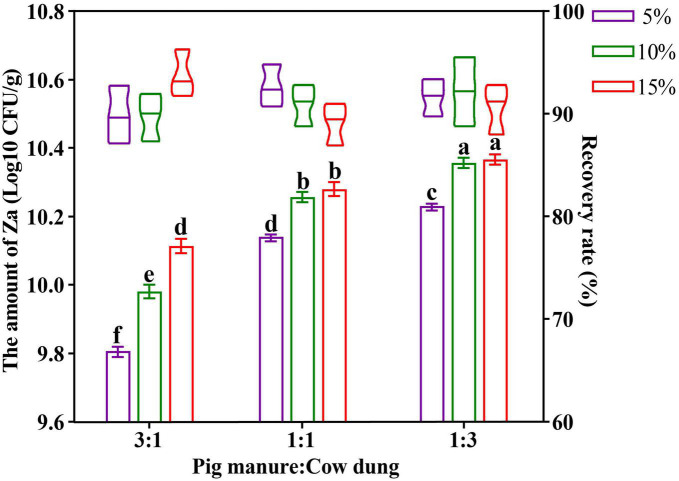
Optimization of organic fertilizer carrier ratio and Za inoculum amount in preparation of solid agents. The abscissa represented the mixing ratio of the organic fertilizer carrier (pig manure: cow dung), the icon (5%, 10%, and 15%) showed the levels of strain Za inoculum, the amount of Za was shown as a bar graph, the recovery rate of Za was shown as the violin plot.

A two-factor and three-level orthogonal test was designed for the carrier mixture ratio and inoculum size of the solid bacterial agent, the test design and results are shown in Supplementary Table 1A. After one-way analysis of variance, the organic fertilizer carrier ratio and Za inoculum amount showed no significant effect on the recovery rate of strain Za in the solid agents. After two-way ANOVA, factor 1 (F = 42.881, *p* = 0.002 < 0.05) and factor 2 (F = 13.185, *p* = 0.017 < 0.05) had significant effects on the amount of Za, and factor 1 was more obvious (Supplementary Table 1B). Based on Figure 1, when the ratio of pig manure and cow dung organic fertilizer in the carrier was 1:3, the amount of strain Za was higher. Simultaneously, the amount of Za increased with the increase in the inoculum amount, but when the inoculum amount reached 15%, there was no significant difference in the amount of strain Za compared with an inoculum amount of 10%. Therefore, the optimal carrier mixture ratio in the preparation of the solid preparation of strain Za was 1:3 (pig manure: cow dung), the optimal inoculum amount of Za was 10%, and the amount of Za in the solid agents could reach 2.34 × 10^10^ CFU/g.

### Quantity dynamics of Za of solid agents with different carriers during the storage period

**FIGURE 2:**
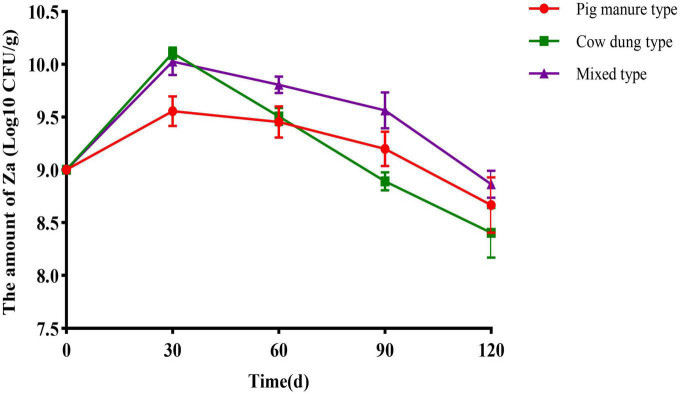
Quantity dynamics of strain Za with different carriers. The inoculation amount of Za bacterial solution was 10%, the error bars indicated standard deviations for three samples.

Pig manure organic fertilizer carrier, cow dung organic fertilizer carrier, and mixed carrier were used to prepare solid agents. At days 30, 60, 90, and 120, samples were collected to detect the number of Za in solid agents, the results are shown in Figure 2. When stored for 30 days, its number in each carrier reached the highest level. Among them, the number of Za contained in the cow dung organic fertilizer carrier reached 1.28 × 10^10^ CFU/g, and that in the mixed carrier was 1.09 × 10^10^ CFU/g. Subsequently, the amount of Za contained in the organic fertilizer carrier decreased gradually. When stored for 120 days, the level of Za contained in the cow dung organic fertilizer carrier was only 2.80 × 10^8^CFU/g. The carrier with the highest level of Za was the mixed carrier, with 7.50 × 10^8^ CFU/g.

### Influence of application amount and mode on the degradation effect of solid agents

**FIGURE 3:**
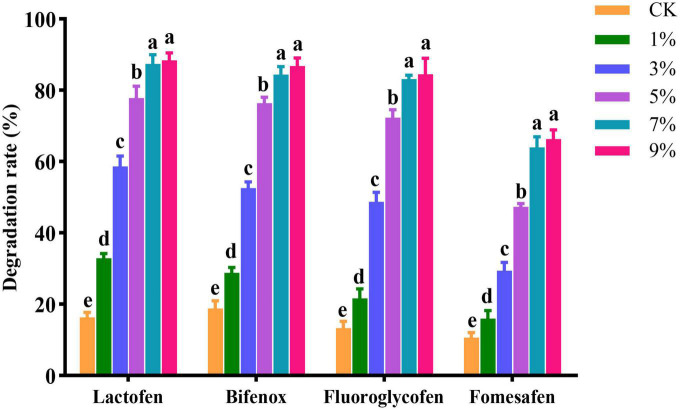
Effect of application rate on degradation of diphenyl ether herbicides by solid agents.

Lactofen, bifenox, fluoroglycofen, and fomesafen herbicides at a concentration of 10 mg/kg dry soil were applied to the soil, and the solid agents were applied in a mixed application amount at 1, 3, 5, 7, and 9%. The concentration of herbicide residues was detected after 4 days. As shown in Figure 3, when the application amount was 1%, the degradation effect of the solid agents was poor. The possible reason was that when the amount of Za in soil was low, the strain showed difficulties in growth and reproduction in a short period. Low biomass of strain Za impeded its effective colonization in the maize rhizosphere, and limited the degradation of herbicide residues. At an application level of 7%, the solid agents could degrade 87.36, 84.40, 83.16, and 63.96% of 10 mg/kg lactofen, bifenox, fluoroglycofen, and fomesafen in soil, respectively, and at 9%, the degradation rates were 88.40, 86.76, 84.47, and 66.32%, respectively. When the application amount was 9%, the degradation effect was not significantly improved compared with 7%. At an application level of 7%, Za could stably colonize the maize rhizosphere and efficiently degrade the residues of diphenyl ether herbicides. Considering the costs and benefits of the solid agent application, the optimal application amount of solid agents was 7%.

**FIGURE 4:**
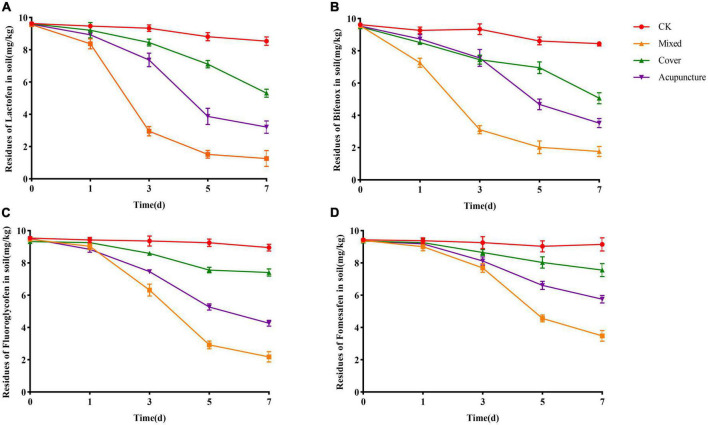
Effects of application mode of solid agents on the degradation of diphenyl ether herbicides. **(A)** Lactofen; **(B)** Bifenox; **(C)** Fluoroglycofen; **(D)** Fomesafen.

The solid agents were applied in three ways, namely covering application, mixed application, and acupoint application. The changes in herbicide residues were detected on the 1st, 3rd, 5th, and 7th days. On the 1st day, the degradation effect of solid agents on herbicide residues in soil was not obvious. The possible reason was that after the solid agents were applied to the soil, they needed to grow and reproduce in soil to form a certain number of scales before the degradation effect was significant. On the 7th day, in the mixed application treatment, the degradation rates of solid agents on lactofen, bifenox, fluoroglycofen, and fomesafen in soil reached 87.40, 82.40, 78.20, and 65.20%, respectively. The degradation effect was better than that achieved *via* acupoint application and covering application. From the perspective of the overall degradation effect, the best application mode was obtained for the mixed application (Figure 4).

### Decrease the phytotoxicity of lactofen to maize by solid agents

**FIGURE 5:**
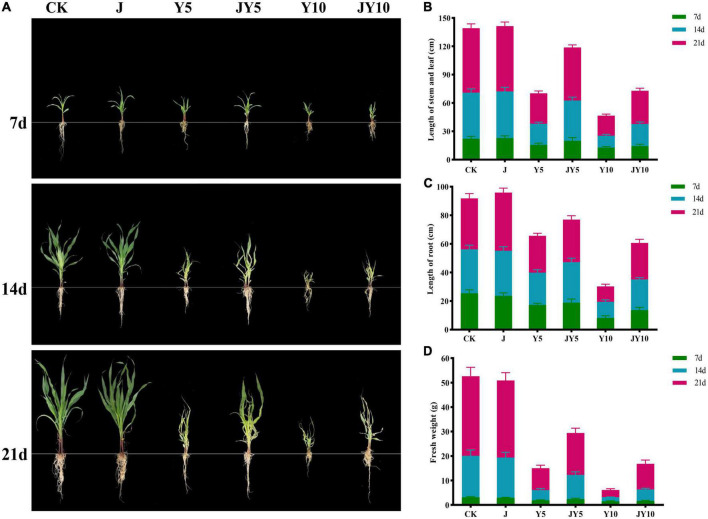
Effects of lactofen and solid agents on physiological indices of maize. **(A)** Growth status of maize under each treatment; **(B)** Length of stem and leaf (cm); **(C)** Length of root (cm); **(D)** Fresh weight (g). CK, control; J, solid agents; Y5, 5 mg/kg lactofen; JY5, 5 mg/kg lactofen + solid agents; Y10, 10 mg/kg lactofen; JY10, 10 mg/kg lactofen + solid agents.

The pot experiment was carried out on the phytotoxicity of lactofen to maize and the removal of phytotoxicity by solid agents at the seedling stage of maize, and the results are shown in Figure 5. The growth indicators of Control and J groups (solid agents) at various maize seedling stages were highly consistent, proving that the application of solid agents did not affect the growth of maize seedlings (Figure 5A). Stem and leaf length, root length, and fresh weight of maize in group Y5 (5 mg/kg lactofen) were 15.6 ± 1.9 cm, 17.3 ± 1.21 cm, and 1.83 ± 0.27 g, whereas in Y10 (10 mg/kg lactofen), they were 12.7 ± 1.06 cm, 8.2 ± 1.51 cm, and 1.45 ± 0.28 g on the 7th day, respectively, which were significantly lower than those of the Control. The difference became more and more significant on the 14th and 21st days. However, the application of solid agents could decrease the phytotoxicity to maize seedlings. The growth indices of JY5 (5 mg/kg lactofen + solid agents) and JY10 (5 mg/kg lactofen + solid agents) were significantly higher than those of Y5 and Y10 after the 7th day of the maize seedling stage. On the 7th day, the growth indices of the maize seedlings in JY10 and Y10 were similar. On the 21st day, the stem and leaf length and root length of Y10 were 21.3 ± 1.69 cm and 10.8 ± 1.57 cm, and those of JY10 were 35.1 ± 2.86 cm and 25.6 ± 2.51 cm, respectively, the fresh weight of maize seedlings in JY10 (10.48 ± 1.53 g) was three times that of Y10 (2.96 ± 0.51 g) (Figures 5B–D). The growth indices of maize seedlings in JY5 were also significantly higher than those in Y5. Based on these results, the application of solid agents could significantly reduce the phytotoxicity to maize caused by the residues of lactofen in soil (Figure 5).

### Colonization observation of strain Za-*gfp* in maize rhizsosphere

**FIGURE 6:**
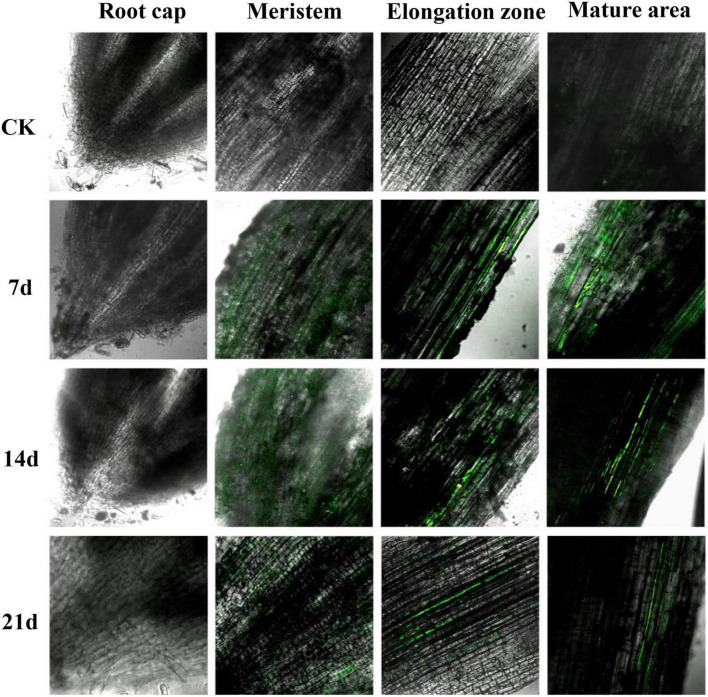
Confocal laser scanning microscope (CLSM) photos of strain Za-*gfp* in solid agents colonized on the root surface of maize seedlings. The experimental materials were obtained from the maize seedling samples of the CK and J groups (solid agents).

Some pesticide-degrading bacteria can colonize the root surface of maize seedlings (Zhang H. et al., 2018; Qian et al., 2022). Based on this, this study explored whether the solid agents could effectively release strain Za-*gfp* in soil and enable its colonization on the surface of maize roots. The root samples at 7, 14, and 21 d were observed under a CLSM. As seen in Figure 6, Za-*gfp* in the solid agents could be effectively released into the plant rhizosphere soil, resulting in the colonization of the root surface. Strain Za-*gfp* mainly colonized the elongation and the mature area of maize root tips, and no colonization was observed in the root cap. The colonization effect was more significant on the 7th day, with a large fluorescence range and a high intensity. A strong green fluorescence could still be observed in the meristem, elongation and mature areas of the maize root system sampled on the 21st day. Most likely, the strain Za-*gfp* in the solid agents could be rapidly released and colonized the maize root system after application to the soil, and the colonization time could exceed 21 days.

### Soil bacterial community structure during the degradation of lactofen by solid agents

**FIGURE 7:**
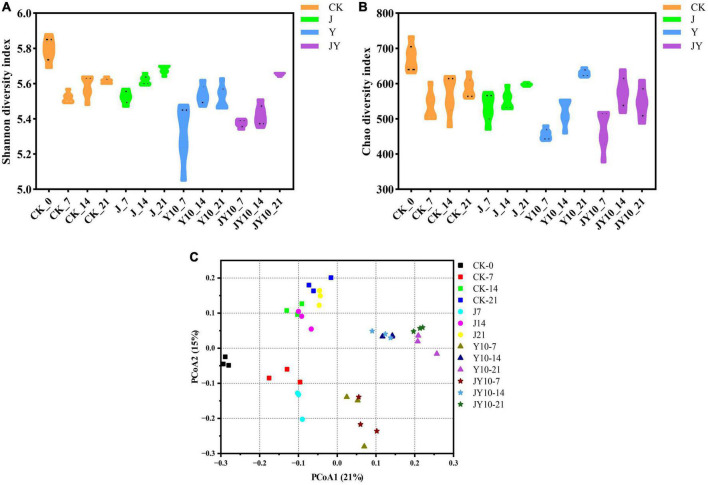
*Alpha* diversity and *Beta* diversity analysis of soil microbial community structure under different treatments. **(A)** Shannon diversity index for microbial communities of different treatments. **(B)** Chao diversity index for microbial communities of different treatments. **(C)** Analysis of the differences between different samples with same treatment condition. CK, no lactofen and solid agents; J, solid agents; Y, lactofen; JY, lactofen and solid agents. Among them, Y10 means 10 mg/kg lactofen, JY10 means 10 mg/kg lactofen and solid agents. The numbers 7, 14, and 21 represented the days of administration, respectively. The Shannon index comprehensively considered the richness and evenness of the community. The larger the Shannon value was, the higher the community diversity was. The Chao index reflected the richness of the community by estimating the number of species in the community, and the larger the index was, the higher the community richness was. PCoA, which could be used to study similarities or differences in sample community composition.

The Shannon index and the Chao index represent the diversity and richness of microbial community, respectively. The *Alpha* diversity of the rhizosphere soil samples from the different treatments showed that the application of 10 mg/kg lactofen to the soil caused a sharp decrease in the diversity of soil bacteria, and the difference reached a highly significant level (Figure 7A). When the solid agents were applied in the presence of 10 mg/kg lactofen, both the Chao and the Shannon index increased significantly, reaching to the level of the Control (Figures 7A,B). The application of solid agents could reverse the adverse effects of herbicide residues on the diversity of the soil bacterial community. The *Beta* diversity showed that the residues of lactofen could change the bacterial community structure, and the treatments with herbicide residues were significantly different from the Control (Figure 7C). The application of solid agents had a certain recovery effect on the soil bacterial community.

**FIGURE 8:**
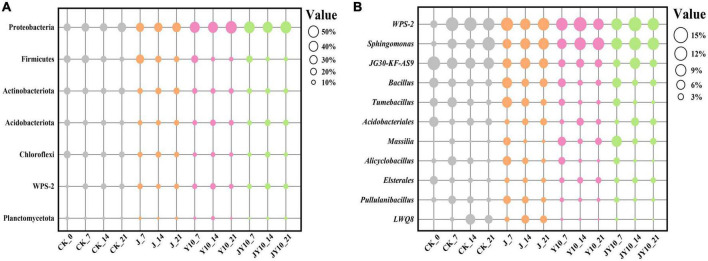
Community composition analysis of soil bacteria at phylum **(A)** and genus **(B)** levels under different treatment conditions. CK, no lactofen and solid agents; J, solid agents; Y10, 10 mg/kg lactofen; JY10 mg/kg lactofen and solid agents. The numbers 7, 14, and 21 represented the days of administration, respectively. Bubble sizes represented relative percentages of different species.

The relative abundances of bacteria at the phylum level under different treatment conditions are shown in Figure 8A. The phyla with relative abundance ratios >0.05 were Proteobacteria, Actinobacteria, Firmicutes, Acidobacteria, and Chloroflexi. Proteobacteria and Firmicutes are the most reported diphenyl ether herbicide-degrading bacteria. The relative abundance of Proteobacteria in groups Y10 and JY10 was significantly higher than that in the Control, leading to the speculation that some strains of Proteobacteria can grow with lactofen as a carbon and nitrogen source. The relative abundances of Proteobacteria in groups J and Control were similar, indicating that the addition of solid agents would not lead to changes in the abundance of Proteobacteria. The relative abundance of Firmicutes in the Control treatment gradually decreased over time, and this change was more pronounced in the Y10 and JY10 groups (Figure 8A). The relative abundance of Firmicutes in the J7 group treatment was the highest among all samples, whereas in groups J and JY, it gradually decreased over time. On the 21st day, the relative abundance of Firmicutes in group J was still significantly higher than that in the other treatments.

The relative abundances of bacterial genera under different treatment conditions are shown in Figure 8B. Genera with a relative abundance ratio >0.04 were *WPS-2*, *Sphingomonas*, *JG30-KF-AS9*, *Bacillus* and *Tumebacillus*. The relative abundance of *Sphingomonas* was significantly higher than that of the Control in each treatment. Its relative abundance increased rapidly after maize planting, and *Sphingomonas* became one of the dominant genera in the rhizosphere soil of maize seedlings. The relative abundance of *Sphingomonas* gradually increased over time, with significantly higher levels in soil containing herbicide residues. It was therefore speculated that some strains of this genus can grow using lactofen in soil, and there may also be strains degrading diphenyl ether herbicides. The abundance of *Bacillus* in the Control gradually decreased over time, which may be related to the cultivation of maize. The relative abundance of *Bacillus* in group J was significantly higher than that in the other treatments, especially in group J7, which had the highest relative abundance of *Bacillus* among all samples. On the 14th day, the relative abundance of *Bacillus* rapidly decreased and remained stable, and on the 21st day, the relative abundance of *Bacillus* in the treatment with solid agents (group J) was significantly higher than that in the other treatments, most likely because strain Za-*gfp* had colonized the rhizosphere of maize (Figure 6) and had become the dominant strain in soil.

## Discussion

Diphenyl ether herbicides have become important herbicides and have been widely studied and applied in agricultural field (Sheu et al., 2006). However, with the large-scale use of diphenyl ether herbicides, the problems of their residues in soil and the toxic effects on organisms have become increasingly prominent (Mukherjee et al., 2007; Wang et al., 2018). Bioremediation is the most promising biotechnological method to eliminate pesticide residue pollution in soil, and some efficient diphenyl ether pesticide-degrading bacteria have been reported, including *Brevundimonas* sp. LY-2 (Liang et al., 2010), *Mycobacterium phocaicum* MBWY-1 (Chen et al., 2011), *Pseudomonas* sp. FB8 (Yang et al., 2011), *Pseudomonas zeshuii* BY-1 (Feng et al., 2012), *Lysinibacillus fluoroglycofenilyticus* cmg86 (Cheng et al., 2015), *Bacillus* sp. FE-1 (Cui et al., 2018), *Edaphocola flava* HME-24. (Hu et al., 2020) and *Bacillus* sp. YS-1 (Shang et al., 2022). The research object of this study, *Bacillus* sp. Za, was a diphenyl ether herbicide-degrading strain isolated in our laboratory (Zhang J. et al., 2018), providing valuable resources for the bioremediation of sites contaminated with diphenyl ether pesticide residues.

The development of microbial degrading agents using highly efficiency strains is widely recognized and considered to be a safe, efficient solution with great application potential (Zhang W. et al., 2020). Compared with other kinds of microbial agents, solid agents are more popular due to their easy preservation, long shelf life, and good remediation effect. The Za solid agents in this study had a viable count of 2.34 × 10^10^ CFU/g on the third day, which remained above 10^8^ CFU/g after 120 days of storage. Within 7 days, the removal rate of diphenyl ether pesticides was about 80% with the application of solid bacterial agents. There have been some reports on the development and application of solid agents, the recovery efficiency of solid agents has been largely improved, and good results have been obtained. For example, starting from the carrier aspect, the effects of kaolinite, montmorillonite, and vermiculite on the efficiency of phenol degradation by *Sphiningomonas* sp. GY2B were evaluated (Gong et al., 2016). From the perspective of the composition ratio of immobilized materials, orthogonal experiments tested the combination of four materials, namely polyvinyl alcohol, sodium alginate, activated carbon, and *Pseudomonas stutzeri* Y2, to stabilize and extend the bacterial degradation of s-triazine herbicides through an immobilization strategy (Zhang B. et al., 2020). At the same time, the remediation effect of bacterial agents on polluted soil was also reflected through a maize growth experiment, which was consistent with the experimental purpose of this study, as shown in Figure 5. Compared with the treatments without solid agents (Y5 and Y10), the herbicide damage to maize seedlings was significantly alleviated with the addition of solid agents (JY5 and JY10), and over time, this phenomenon was more obvious. The CLSM photos of *Bacillus* sp. Za-*gfp* in solid agents on the root surface of maize seedlings indicated that the diphenyl ether herbicide-degrading bacteria Za-*gfp* could colonize the root surface of maize seedlings and further proved that the development of solid agents could promote the remediation of sites contaminated with diphenyl ether herbicides. By evaluating the influence of different external conditions (pH, temperature, etc.) on solid agents, the storage time of solid agents was extended from the perspective of environmental factors (More et al., 2015). Various studies have shown that the ratio of different strains and different carrier materials can affect the storage time of solid agents (Sayed and Behle, 2017b), which is in agreement with the findings of the present study. Moreover, the degradation effect of the solid agents on the four diphenyl ether herbicides also reached an ideal level, providing a suitable microbial agent for the removal of the diphenyl ether herbicides.

Both environmental pollutants and the use of microbial solid agents can alter the structure and diversity of the microbial communities. For example, long-term metal and radionuclide contamination affected the structure and function of microbial communities (Rogiers et al., 2021). The addition of synthetic and biological pesticides would directly affect the total bacterial community diversity in the rhizosphere soil (Walvekar et al., 2017). Microbial solid agents need to be prepared by selecting suitable materials as carriers, and the addition of these solid agents will also change the microbial community structure. In a previous study, an enhanced nitrogen removal from brine wastewater by adding biochar-immobilized bacteria was observed, and the biochar bacterial agent had a significant effect on the microbial community structure (Zhao et al., 2022). In another study, the highly efficient degrading bacterial strain *Daracoccus aminovorans* HPD-2 significantly promoted the degradation of Polycyclic Aromatic Hydrocarbons (PAHs) in soil, increased the abundance of embedded stratified bacteria in soil and enhanced the potential indigenous degrading bacteria at the same time (Ren et al., 2022). The carrier material selected in this study was organic fertilizer (pig manure and cow dung), and the mixing ratio of these two types of carriers was optimized to 1:3. The microbial community structure and biodiversity results showed that the residues of the pesticide lactofen reduced the soil bacteria diversity, and the application of solid agents could restore this diversity. Based on the analysis of the relative abundance of species at the bacterial phylum and genus levels, the relative abundance of Proteobacteria was significantly increased in the treatment with the addition of lactofen residues, most likely because the herbicide residues changed the soil environment and the bacterial preference. *Sphingomonas* quickly gained the dominant species status after maize planting, indicating that some strains of this genus may be involved in the process of cultivating soil bacterial communities in maize. Its relative abundance was higher in soil containing herbicide residues, leading to the assumption that there are strains of this genus that can degrade diphenyl ether herbicides. Various research results showed that environmental pollutants and microbial agents can alter microbial communities and biodiversity (Satapute et al., 2019; Giambò et al., 2022). However, the soil response to microbial preparations and the mechanism of action in soil rhizosphere are still unclear. Nevertheless, relying on modern molecular biotechnology and bioinformatics methods, it is possible to deeply explore the mechanism of action of microbial preparations.

In this study, the development and performance parameters of the solid degrading microbial agent were determined under laboratory conditions. Complex environmental factors represent a major obstacle to the production and application of solid microbial agents. Although this study applied multi-factor optimization for the development of solid agents (such as organic fertilizer mixing ratio, inoculum amount, and application mode), for practical applications, many factors still need to be considered for further optimization. At the same time, the mechanism of action of microbial agents in soil and rhizosphere environments needs to be further explored.

## Data availability statement

The original contributions presented in this study are included in the article/Supplementary material, further inquiries can be directed to the corresponding author.

## Author contributions

GZ designed the work, conducted the experiments, and wrote the manuscript. YT and HY participated in revising the manuscript. JL and DM conducted the experiments and analyzed the data. XH and RF guided the data analysis and revised the manuscript. All authors contributed to the study and approved the final submitted version.
