# Supplementary material for: Development of solid agents of the diphenyl ether herbicide degrading bacterium Bacillus sp. Za based on a mixed organic fertilizer carrier
Source: Front Microbiol. 2022 Nov 24;13:1075930. doi: 10.3389/fmicb.2022.1075930 (PMC9729343; doi:10.3389/fmicb.2022.1075930)
Supplement: Supplementary file 1 [file Table_1.docx]

**Supporting Information for**

**Development of solid agents of the diphenyl ether herbicide degrading bacterium *Bacillus* sp. Za based on a mixed organic fertilizer carrier**

Guoqiang Zhao^1^, Yanning Tian^1^, Houyu Yu^1^, Jintao Li^1^, Dongmei Mao^1^, Rayan Mazin Faisal^2^, Xing Huang^1*^

^1^Department of Microbiology, College of Life Sciences, Nanjing Agricultural University, Nanjing, China

^2^ Department of biology, College of Science, University of Mosul, Mosul, Iraq

*Corresponding author: Xing Huang

E-mail: huangxing@njau.edu.cn

**SUPPLEMENTARY MATERIAL**

Table 1A Orthogonal table of factor combination for optimization of bacterial preparation

| Number | Factor 1 | Factor 2 | 3d amount of Za  （Log10 CFU/g） | Recovery rate  (%) |
| --- | --- | --- | --- | --- |
|  | Mixing ratio  （pig manure∶cow dung） | Inoculum  (%) |  |  |
| 1 | 3∶1 | 5 | 9.81 | 89.87±2.32 |
| 2 | 3∶1 | 10 | 9.98 | 89.79±1.91 |
| 3 | 3∶1 | 15 | 10.11 | 93.75±1.91 |
| 4 | 1∶1 | 5 | 10.14 | 92.66±1.69 |
| 5 | 1∶1 | 10 | 10.26 | 90.97±1.67 |
| 6 | 1∶1 | 15 | 10.28 | 89.14±1.69 |
| 7 | 1∶3 | 5 | 10.23 | 91.65±1.49 |
| 8 | 1∶3 | 10 | 10.36 | 92.19±2.76 |
| 9 | 1∶3 | 15 | 10.37 | 90.69±2.02 |

Table 1B Results of two-way ANOVA for optimization of bacterial preparation

| Source of difference | Sum of squares | Degrees of freedom | Mean square | F value | p value |
| --- | --- | --- | --- | --- | --- |
| Mixing ratio | 0.197 | 2 | 0.099 | 42.881 | 0.002** |
| Inoculum | 0.061 | 2 | 0.030 | 13.185 | 0.017* |
| Residual | 0.009 | 4 | 0.002 |  |  |

Note：R^2^=0.966；**p*< 0.05 ；***p*< 0.01
